# Colistin Treatment Outcomes in Gram-Negative Bacterial Infections in the Northeast of Romania: A Decade of Change Through Pandemic Challenges

**DOI:** 10.3390/antibiotics14030275

**Published:** 2025-03-07

**Authors:** Madalina Alexandra Vlad, Luminita Smaranda Iancu, Olivia Simona Dorneanu, Alexandru Duhaniuc, Mariana Pavel-Tanasa, Cristina Gabriela Tuchilus

**Affiliations:** 1 Department of Microbiology, Faculty of Medicine, “Grigore T. Popa” University of Medicine and Pharmacy, 700115 Iasi, Romania; madalina.vlad@umfiasi.ro (M.A.V.); luminita.iancu@umfiasi.ro (L.S.I.); olivia.dorneanu@umfiasi.ro (O.S.D.); alexandru.duhaniuc@umfiasi.ro (A.D.); cristina.tuchilus@umfiasi.ro (C.G.T.); 2 Medical Analysis Laboratory, “Saint Spiridon” County Clinical Emergency Hospital, 700111 Iasi, Romania; 3 Department of Immunology, Faculty of Medicine, “Grigore T. Popa” University of Medicine and Pharmacy, 700115 Iasi, Romania; 4 Laboratory of Immunology, “Saint Spiridon” County Clinical Emergency Hospital, 700111 Iasi, Romania

**Keywords:** colistin resistance, Gram-negative bacteria, SARS-CoV-2 pandemic, multidrug-resistant infections, antimicrobial stewardship, mortality

## Abstract

**Background**: The rise in multidrug-resistant (MDR) Gram-negative bacteria (GNB) poses a critical threat to global health, exacerbated by the increased use of antibiotics during the SARS-CoV-2 pandemic. Colistin, often considered a last-resort antibiotic, has seen heightened usage, raising concerns about resistance and clinical outcomes. This study investigates the evolution of colistin resistance in GNB during the SARS-CoV-2 pandemic, with a focus on clinical outcomes in colistin-treated patients. **Methods**: Conducted in “Saint Spiridon” Hospital, Iasi, Romania, the study assesses antibiotic usage, trend in GNB resistance to colistin, and mortality rates across distinct pandemic phases (pre-pandemic, Delta, Omicron, and post-pandemic). A retrospective longitudinal analysis spanning 2011 to 2023 was performed. Statistical models were employed to analyze mortality risk and assess the pandemic’s impact on antimicrobial dynamics. **Results**: Colistin usage increased significantly during the pandemic, with the highest doses recorded during the Omicron phase. Resistance to colistin, particularly among *A. baumannii* and *K. pneumoniae*, was associated with increased mortality rates. The statistical model demonstrated robust predictive accuracy for mortality across the pandemic phases, with an AUC of 0.866 in the post-pandemic period. The findings underscore the critical role of *A. baumannii* as a driver of adverse outcomes, with co-infections significantly linked to systemic infections and higher mortality. **Conclusions**: The study highlights the evolving trends in colistin therapy and resistance, emphasizing the pandemic’s impact on antimicrobial stewardship and the persistent challenges posed by MDR pathogens. We emphasize the need for antimicrobial resistance surveillance, including the monitoring of colistin resistance, which is considered a last-resort antibiotic.

## 1. Introduction

The SARS-CoV-2 pandemic has significantly heightened the antimicrobial resistance (AMR) crisis, increasing the pressure on the healthcare and social care systems [1]. According to a 2022 CDC report, antimicrobial-resistant infections caused over 29,400 deaths in 2020 in United States (U.S.), with nearly 40% of these infections acquired in hospital settings. The pandemic’s impact on healthcare resources, marked by an increased use of medical devices, prolonged patient hospitalizations, staffing shortages, and compromised infection control measures, has driven a surge in healthcare-associated infections [2].

The World Health Organization (WHO) has identified AMR, particularly among Gram-negative species, as a growing global health threat [3].

The SARS-CoV-2 pandemic in 2020 created an opportunity to reduce antibiotic consumption, as the strict public health measures, including lockdowns, social distancing, and improved hygiene practices, led to a decrease in bacterial co-infections and overall healthcare utilization in many countries [4,5]. Additionally, the growing awareness of viral versus bacterial infections during the pandemic highlighted the need for more prudent antibiotic use, and many countries have reported reductions in antibiotic prescriptions, particularly for respiratory infections [6]. However, this potential was not fully realized in Romania, where antibiotic consumption remained high, likely due to empirical prescriptions in COVID-19 cases, self-medication, and gaps in antimicrobial stewardship programs [7]. Thus, an increased use of broad-spectrum antibiotics, such as penicillins with inhibitors, extended-spectrum cephalosporins, carbapenems, and reserve antibiotics like colistin was registered during the COVID-19 pandemic years in Romania [8]. A comparison of EARS-Net data from the pre-pandemic (2017) and post-pandemic (2021) periods revealed an increasing trend in AMR, particularly for *Klebsiella pneumoniae* and *Acinetobacter baumannii* isolates in Romania and other southern and eastern European regions [9]. This upward trend underscores the heightened risk of multidrug-resistant (MDR) pathogens that emerged during the COVID-19 pandemic.

Antimicrobial resistance in *K. pneumoniae* has emerged as a major concern in recent years [10,11]. Resistance to carbapenems is particularly concerning due to its association with cross-resistance to multiple other antibiotic classes, which greatly restricts treatment options and leads to increased rates of treatment failure [12]. In those cases, last-resort antibiotics such as colistin are frequently used in combination with other antibiotics [13,14]. However, the growing global prevalence of carbapenem-resistant and MDR *K. pneumoniae* has led to increased dependence on colistin in recent years, which has, in turn, contributed to the rise in colistin-resistant strains [10].

Before the emergence of the SARS-CoV-2 pandemic, colistin resistance was already recognized as a growing global health concern, primarily driven by the overuse of colistin in both human medicine and animal agriculture. The increasing reliance on colistin as a last-resort antibiotic for carbapenem-resistant Gram-negative bacterial (GNB) infections has led to the gradual selection of resistant strains, complicating treatment strategies for multidrug-resistant (MDR), extensively drug-resistant (XDR), and pan-drug-resistant (PDR) infections [15].

A global study on colistin resistance revealed that *K. pneumonaie* had the highest resistance rate among isolates in 2020, reaching 12.9% (4 out of 31 isolates) compared to approximately 2.9% in previous years [16]. A meta-analysis on *Pseudomonas aeruginosa* reported an increase in the overall colistin resistance rate from 2% in the period of 2006–2010 to 5% in the period of 2020–2023 [17]. A cross-sectional study performed in Egypt in the period of 2022–2023 found colistin resistance rates of 12.3% in *K. pneumoniae*, 7% in *P. aeruginosa*, and 73.7% in *A. baumannii* [18]. These findings underscore the escalating challenge of colistin resistance in critical healthcare settings, especially since 2020. Carbapenems are among the last remaining options for treating *A. baumannii* infections [19]. Importantly, the increasing prevalence of carbapenem-resistant strains led the World Health Organization (WHO) to designate carbapenem-resistant *A. baumannii* as a “Priority 1 critical pathogen” for antibiotic research in its 2017 report, issued a few years before the onset of the COVID-19 pandemic [20]. Colistin, a last-resort antibiotic, which was abandoned in the 1980s due to its nephrotoxic and neurotoxic side effects, has been reintroduced to combat the growing threat of carbapenem-resistant GNB [21]. However, the reintroduction of colistin led to a rise in the prevalence of colistin-resistant strains over the past decade [20]. The COVID-19 pandemic further exacerbated the antimicrobial resistance crisis, likely due to the widespread use of broad-spectrum antibiotics, including colistin, to treat secondary bacterial infections in critically ill COVID-19 patients. Many hospitals reported a surge in multidrug-resistant infections due to extended hospitalization days, invasive mechanical ventilation, and excessive antibiotic prescriptions during the pandemic [22]. *A. baumannii*, in particular, became a major concern in COVID-19 wards, with studies showing alarmingly high colistin resistance rates of 52% in an Iranian study [23] or 91.2% in Wuhan [24].

According to the EARS-Net surveillance study, colistin resistance in *P. aeruginosa* increased from 1% to 4% across Europe in the period of 2013–2016 [25]. In 2016, the majority of colistin-resistant isolates from Europe were reported in Italy and Greece. In China, the colistin resistance rates have been reported to range between 1 and 7% [26,27].

The COVID-19 pandemic significantly disrupted healthcare systems and practices, leading to the reallocation of antimicrobial stewardship resources to support the overwhelmed healthcare workforce. During the peak of the crisis, this shift reduced the capacity to implement antimicrobial stewardship initiatives and provide guidance on the rational use of antibiotics in COVID-19 patients [28]. The urgent focus on clinical care, combined with limited healthcare resources, contributed to an increased risk of inappropriate antimicrobial prescribing [5,29,30].

Colistin resistance is relatively uncommon in clinical isolates of *Escherichia coli* compared to *A. baumannii* and *K. pneumoniae*. Between 2010 and 2014, resistance rates were reported at 0.2% in clinical isolates and 0.9% in commercial meat samples. In Taiwan, *E.coli* resistance rates ranged from 1.1% to 8.7% between 2012 and 2015 [31].

The aim of the study was to investigate the evolution of colistin resistance in Gram-negative bacteria (GNB) during the SARS-CoV-2 pandemic, as well as in the pre-pandemic and post-pandemic periods, with a focus on the clinical outcomes of colistin-treated patients. Conducted in a hospital in the northeast of Romania, “Saint Spiridon” County Clinical Emergency Hospital, Iasi, the study seeks to assess the pandemic’s influence on colistin usage, resistance patterns, and mortality rates across distinct pandemic phases (pre-pandemic, Delta, Omicron, and post-pandemic) while also completing the overall picture of colistin resistance.

## 2. Results

The patient population shows fluctuations over time, peaking in 2014 (502 patients) and declining sharply in 2020 (247 patients), likely reflecting the COVID-19 pandemic’s impact on hospital admissions (Table 1).

Males consistently form a higher proportion of the patients (around 60% annually), while females account for approximately 40%.

The median age remains stable, ranging between 63 and 66 years, indicating that these infections predominantly affect older adults.

**Figure 1:**
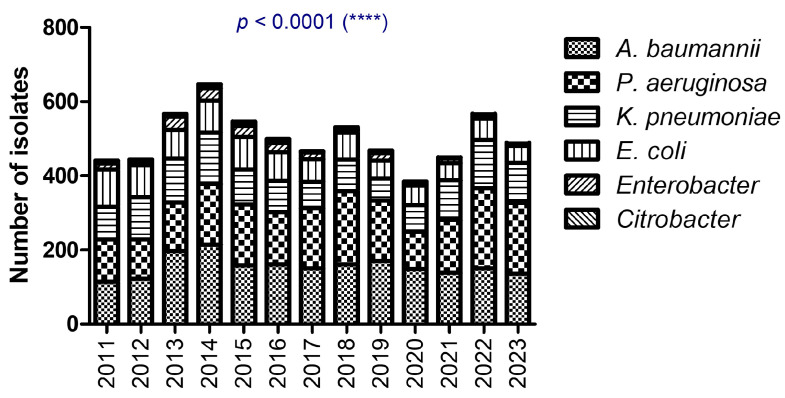
The differences in isolate distribution between 2011 and 2023 (**** *p* < 0.0001; chi-squared test).

The distribution of bacterial isolates (*A. baumannii*, *P. aeruginosa*, *K. pneumoniae*, *E. coli*, and others) varied over the study period from 2011 to 2023. Figure 1 captures the trends in infection patterns and highlights significant shifts in pathogen prevalence over time, evaluated using chi-squared statistical tests (Figure 1). While the total number of *A. baumannii* isolates did not change before and after the pandemic, there was a relative decrease in *P. aeruginosa* isolates in 2020.

**Figure 2:**
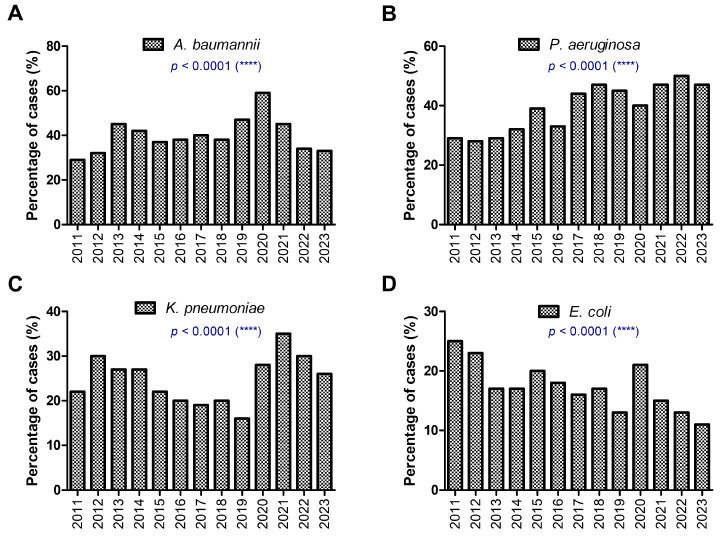
The differences in GNB distribution in colistin-treated patients between 2011 and 2023. (**A**) *A. baumannii*; (**B**) *P. aeruginosa*; (**C**) *K. pneumoniae*; (**D**) *E. coli* (**** *p* < 0.0001; chi-squared test).

The percentage distribution of GNB in colistin-treated patients over the period of 2011 to 2023 is illustrated, highlighting temporal trends in Figure 2. Each sub-panel (A–D) focuses on a specific GNB species (*A. baumannii*, *P. aeruginosa*, *K. pneumoniae*, and *E. coli*), with statistical significance (*p* < 0.0001) derived from chi-squared tests, indicating important variations and potential shifts in prevalence or treatment efficacy over time (Figure 2). The highest percentage of *A. baumannii* isolates was registered in 2020 (Figure 2A), while *P. aeruginosa* prevalence showed a generally increasing trend from 29% in 2011 to 47% in 2023 (Figure 2B). Interestingly, the proportion of *K. pneumoniae* isolates showed a decreasing trend from 2012 (30%) to 2019 (16%), which increased again starting with 2020, reaching a value over 28% (Figure 2C). The increase in the other isolates was accompanied by a reduction in the percentage of *E. coli* strains over the study period from 25% in 2011 to 11% in 2023, with an extra peak of 21% in 2020 (Figure 2D).

**Figure 3:**
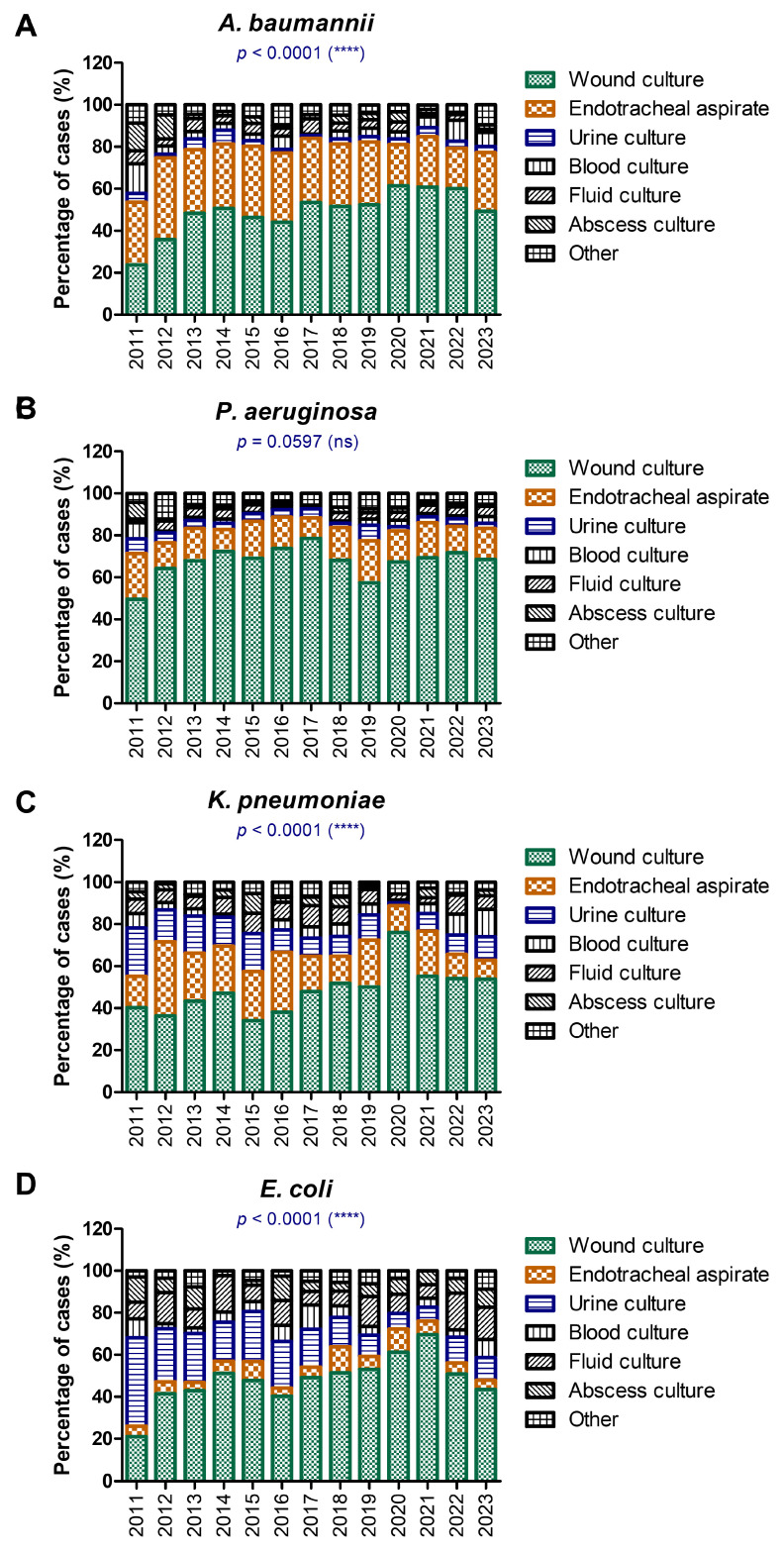
The differences in sample types between 2011 and 2023 for each isolated GNB. (**A**) *A. baumannii*; (**B**) *P. aeruginosa*; (**C**) *K. pneumoniae*; (**D**) *E. coli* (**** *p* < 0.0001, ns—not significant; chi-squared test).

The distribution of sample types, from which various GNB were isolated between 2011 and 2023, are depicted in Figure 3. Each subpanel (A–D) focuses on a specific pathogen (*A. baumannii*, *P. aeruginosa*, *K. pneumoniae*, and *E. coli*), showing trends across different sample sources, including wound cultures, endotracheal aspirates, urine cultures, blood cultures, fluid cultures, abscess cultures, and others (Figure 3).

The majority of *A. baumannii* isolates were obtained from wound cultures and endotracheal aspirates. The increasing trend in wound culture isolates emphasizes their role in surgical site infections and trauma cases (Figure 3A).

*P. aeruginosa* isolates showed a balanced distribution across the years, with a high predominance in wound cultures and endotracheal aspirates (Figure 3B). This stability trend might reflect *P. aeruginosa*’s adaptability to multiple anatomical sites, including wounds, the respiratory tract, and urinary systems. Its role in chronic infections, such as in patients with cystic fibrosis or chronic wounds, may explain the lack of significant fluctuations. A steady dominance of wound cultures was followed by blood and urine cultures.

*K.pneumoniae* isolates showed a visible rise in wound cultures in 2020 (of 76.05%) when compared to the previous 3 years (in average 49.88%) or the following 3 years (54.34%), Figure 3C. Interestingly, there was a noticeable increase in blood cultures from less than 6% before the COVID-19 pandemic to over 10% in 2022 with a maximum of 12.96% in 2023. The rise in blood culture isolates reflected the growing burden of bloodstream infections caused by *K. pneumoniae*, especially multidrug-resistant strains. Its presence in diverse sources highlights its versatility as both a community-acquired and nosocomial pathogen.

Besides being isolated from wound cultures, *E. coli* showed the highest prevalence in urine cultures compared to the other GNB, consistent with *E. coli*’s role as a leading cause of urinary tract infections (UTIs). However, a gradual decline in isolates from urine cultures is observed over the years, coupled with slight increases in other sources, such as blood and wound cultures, possibly due to a rising in cases of invasive *E. coli* infections or improved detection methods in hospital settings (Figure 3D).

**Figure 4:**
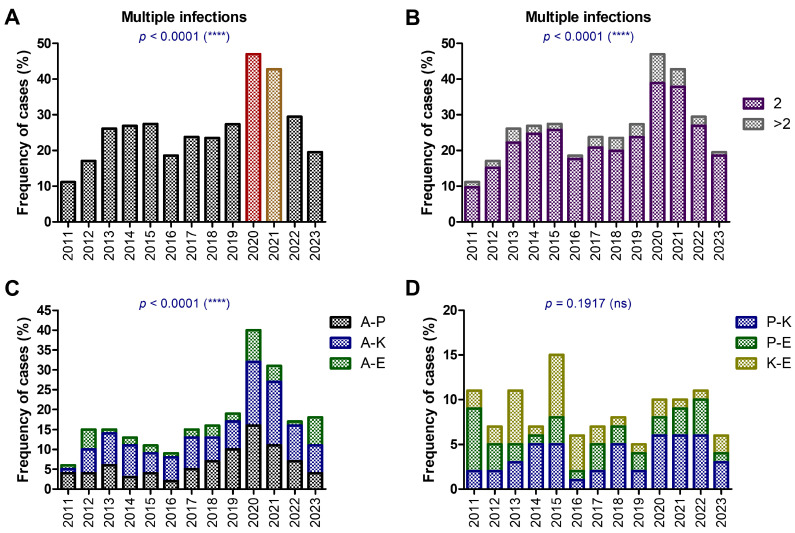
The frequency of multiple GNB infections between 2011 and 2023. (**A**) Frequency of cases with at least two GNB co-infections. The highest frequency is highlighted in red and the second highest in brown. (**B**) Frequency of two (purple bars) or more (gray bars) co-infections. (**C**) Frequency of co-infection of *A. baumannii* with *P. aeruginosa*, *K. pneumoniae*, or *E. coli*. (**D**) Frequency of co-infection of *P. aeruginosa* with *K. pneumoniae or E. coli*, as well as co-infection of *K. pneumoniae* with *E. coli* (**** *p* < 0.0001, ns—not significant; chi-squared test).

Trends in the frequency of multiple GNB infections between 2011 and 2023 are illustrated in Figure 4. The data are divided into four panels (A–D), each focusing on different co-infection types or combinations. There is a notable increase in the frequency of multiple infections during certain years, peaking significantly in 2020–2021 (*p* < 0.0001). The spike in co-infections during these years (over 42%) may reflect changes in clinical practices, bacterial resistance patterns, or increased antibiotic use, potentially linked to the pandemic-related stress on healthcare systems (Figure 4A). In only a few cases, counting for less than 8%, more than two GNB were isolated (Figure 4B). In 2020, the majority of multiple infection cases (85%) were positive for *A. baumannii* (Figure 4C), with the rest being positive for either *P. aeruginosa*, *K. pneumoniae*, or *E. coli* (Figure 4D).

**Figure 5:**
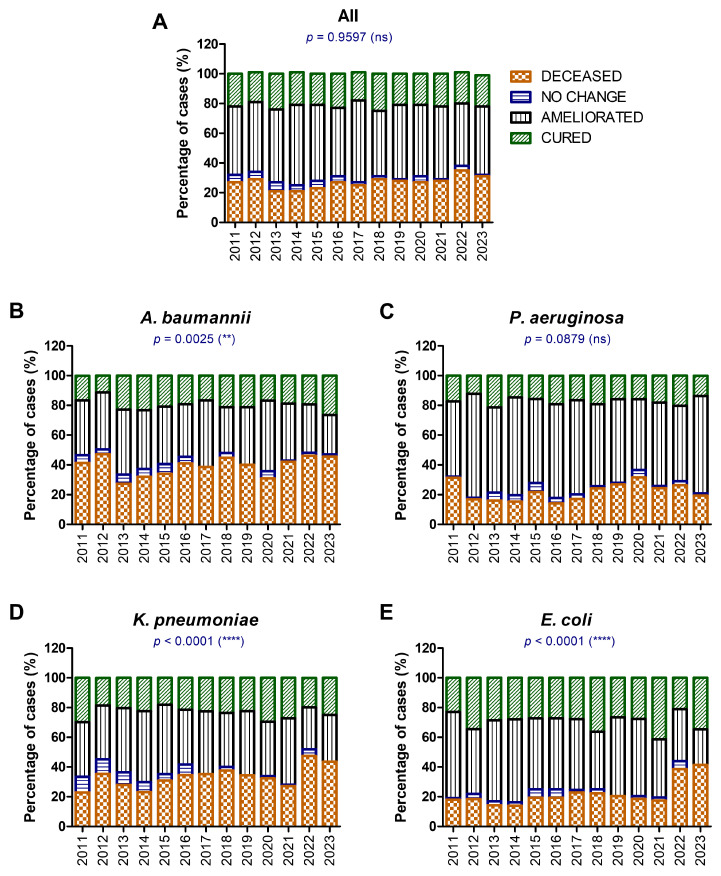
The differences in mortality rates between 2011 and 2023 for (**A**) all cases of GNB infections; (**B**) *A. baumannii*; (**C**) *P. aeruginosa*; (**D**) *K. pneumoniae*; and (**E**) *E. coli* (** *p* < 0.01, **** *p* < 0.0001, ns—not significant; chi-squared test).

The mortality outcomes for patients with GNB infections from 2011 to 2023 are presented in Figure 5. The data are broken down into overall mortality outcomes (Panel A) and specific outcomes for individual pathogens, including *A. baumannii* (Panel B), *P. aeruginosa* (Panel C), *K. pneumoniae* (Panel D), and *E. coli* (Panel E). Each bar represents the percentage distribution of cases by outcome, namely deceased, no change, ameliorated, or cured. Statistical significance for changes in mortality rates over time is calculated using chi-squared tests.

Mortality rates (deceased cases) remained stable over time, with minor fluctuations. The proportion of cases with “cured” or “ameliorated” outcomes also remains largely consistent (Figure 5). The lack of significant variation suggests stable treatment outcomes for GNB infections overall, possibly due to a consistent use of last-resort antibiotics like colistin.

For *A. baumannii*, mortality rates show a statistically significant increase over time (*p* = 0.0025), particularly during and after 2017. This trend aligns with the pandemic years (2019–2021), where hospitalization days and ventilator use likely increased *A. baumannii*-related infections. For *P. aeruginosa*, mortality rates appear stable, with minor increases after 2015, but overall changes are not statistically significant. *P. aeruginosa* remains a persistent pathogen but does not show the same worsening outcomes as *A. baumannii*, likely due to its slightly better susceptibility to treatment options.

For *K. pneumoniae*, mortality rates exhibit a sharp increase during the pandemic years, with significant shifts in outcomes observed. The percentage of “cured” cases decreases, reflecting a worsening overall prognosis for *K. pneumoniae* infections. The rise in mortality rates aligns with the spread of carbapenem-resistant *K. pneumoniae* (CRKP), necessitating colistin use.

For *E. coli*, mortality rates remain low compared to other pathogens, though significant differences are observed (*p* < 0.0001). A gradual decline in cured cases suggests a slight worsening of outcomes over time. In terms of statistical significance (*p* < 0.0001), despite lower mortality, the significant differences reflect subtle changes in treatment efficacy or patient populations.

Since the COVID-19 pandemic years of 2020–2021 provoked the above-mentioned shifts in GNB distribution, we next analyzed the frequency of GNB infections treated with colistin during four distinct periods—pre-pandemic, Delta pandemic, Omicron pandemic, and post-pandemic (2017–2023)—which is presented over the analyzed timeline. Each sub-panel highlights trends for specific pathogens, including *A. baumannii* (Panel A), *P. aeruginosa* (Panel B), *K. pneumoniae* (Panel C), and *E. coli* (Panel D). The statistical significance of differences across these periods is assessed using chi-squared tests.

The frequency of *A. baumannii* infections increased significantly during the Delta pandemic phase but declined during the Omicron and post-pandemic phases. The peak during Delta reflects the surge in *A. baumannii*-related infections, often associated with ventilator-associated pneumonia (VAP) and prolonged hospitalization days. The rise during the Delta period highlights the strain on healthcare systems, with increased hospitalization days and invasive procedures contributing to a spike in *A. baumannii* infections. The subsequent decline might suggest an improvement in the infection control and patient management in later phases.

The frequency of *P. aeruginosa* infections remained relatively stable across all periods, with no significant fluctuations. The stability reflects *P. aeruginosa*’s role as a persistent nosocomial pathogen that consistently impacts critically ill patients, regardless of external factors like the pandemic.

The frequency of *K. pneumoniae* infections increased significantly during the Delta and Omicron pandemic phases, with a subsequent decline post-pandemic. Peaks during Delta and Omicron reflect the pathogen’s growing resistance, especially in patients with bloodstream and respiratory infections.

**Figure 6:**
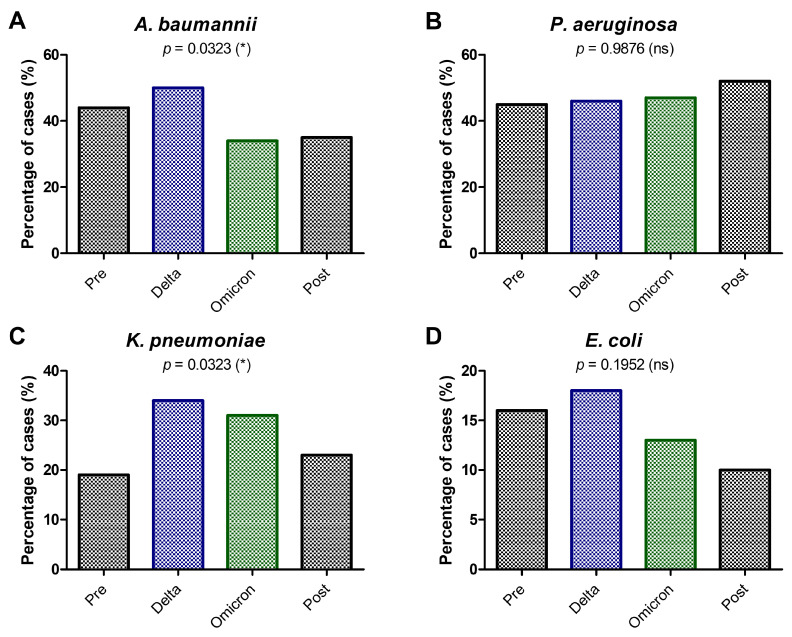
Frequency of GNB infections treated with colistin between 2017 and 2023 during the pre-pandemic, COVID-19 pandemic (Delta, Omicron), and post-pandemic periods. (**A**) *A. baumannii*; (**B**) *P. aeruginosa*; (**C**) *K. pneumoniae*; (**D**) *E. coli* (* *p* < 0.05, ns—not significant; chi-squared test).

The frequency of *E. coli* infections showed a minor increase during Delta but decreased in the Omicron and post-pandemic phases. *E. coli* plays a smaller role in colistin-treated infections, as it is more commonly associated with community-acquired rather than nosocomial infections. Statistical significance (*p* = 0.1952, not significant) indicates no significant differences in the frequency of *E. coli* infections across the study periods. The minor increase during Delta may reflect healthcare disruptions or the inclusion of more severely ill patients during the pandemic. The general stability aligns with *E. coli*’s reduced reliance on colistin compared to other pathogens (Figure 6).

Overall, Figure 6 highlights the dynamic shifts in GNB infections treated with colistin across pandemic phases. The data emphasize the significant burden of *A. baumannii* and *K. pneumoniae* during pandemic peaks, correlating with increased hospitalization days and invasive procedures in our hospital over the course of the analyzed period.

**Figure 7:**
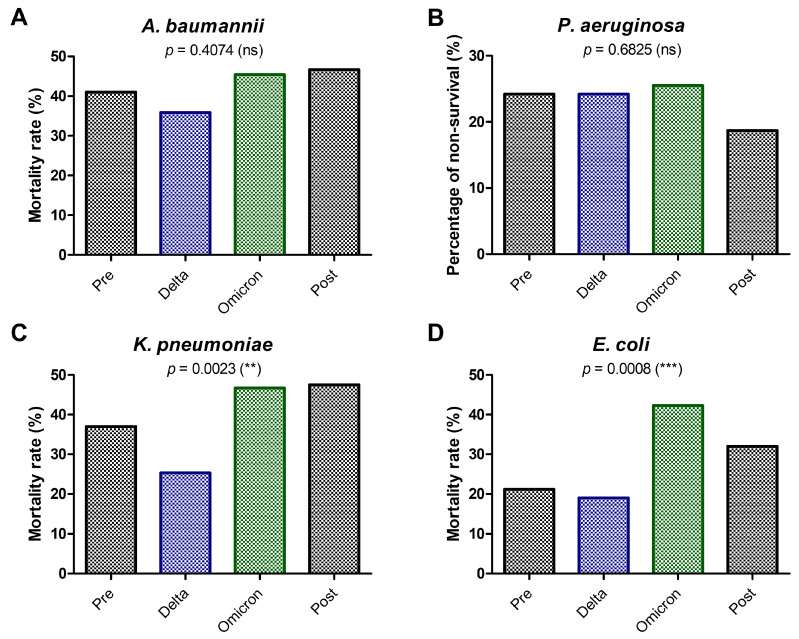
Mortality rate for GNB infections treated with colistin between 2017 and 2023 during the pre-pandemic, COVID-19 pandemic (Delta, Omicron), and post-pandemic periods. (**A**) *A. baumannii*; (**B**) *P. aeruginosa*; (**C**) *K. pneumoniae*; (**D**) *E. coli* (** *p* < 0.01, *** *p* < 0.001, ns—not significant; chi-squared test).

The persistent mortality burden of GNB infections treated with colistin during the pandemic phases is highlighted in Figure 7. Despite changes in the *A. baumannii* frequency over the pandemic phases, no statistically significant differences in mortality rates were noticed (Figure 7A), with similar data for *P. aeruginosa* (Figure 7B). Those stable mortality rates for *A. baumannii* and *P. aeruginosa* might reflect their persistent lethality and resistance patterns regardless of the pandemic phase. However, a notable increase in the mortality rates during the Omicron phase was observed for *K. pneumoniae* (Figure 7C) and *E. coli* (Figure 7D), possibly due to the healthcare system strain and delays in treatment during the pandemic’s peak. These results might highlight the continuous need for enhanced infection control measures, novel antibiotics, and sustained surveillance to address these resistant pathogens.

The lack of significant reductions in mortality for most pathogens suggests that challenges in enhancing colistin efficacy or resistance control persisted over time. The consistently high mortality rates, exceeding 45% for *A. baumannii* and *K. pneumoniae*, underline the importance of exploring and implementing alternative treatment options to improve patient outcomes.

**Figure 8:**
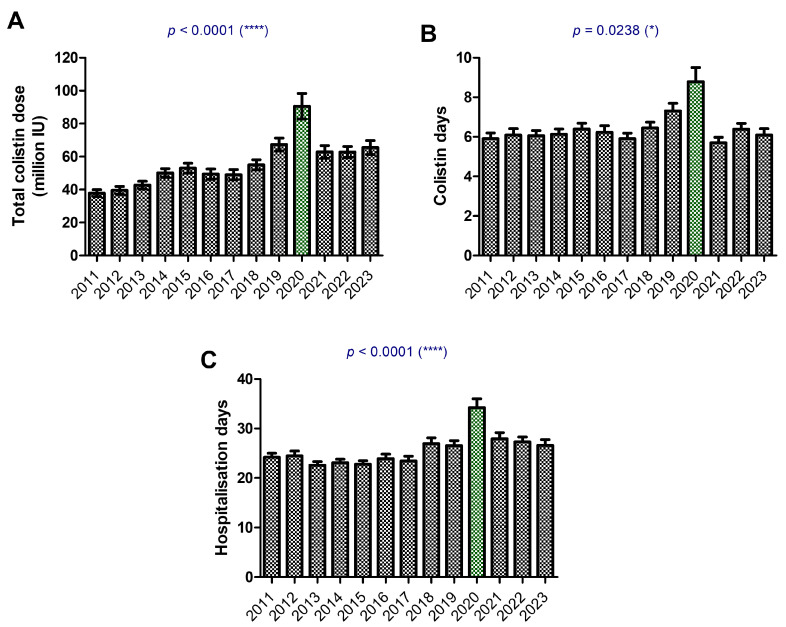
Colistin treatment between 2011 and 2023. (**A**) Colistin number of vials during hospitalization expressed in million IU; (**B**) days of colistin treatment; (**C**) hospitalization days. Bars represent the mean ± s.e.m (* *p* < 0.05, **** *p* < 0.0001; Kruskal–Wallis with Dunn’s multiple comparison test). The green bars indicate the highest values.

The evolving trends in colistin therapy demonstrate increased use during the COVID-19 pandemic and underscore the ongoing challenge of MDR infections in the post-pandemic period. Colistin doses, expressed in million IU administered over the hospitalization period, steadily increased over the years, peaking sharply in 2020 during the COVID-19 pandemic. Following 2020, colistin doses stabilized but remained higher than pre-2020 levels (Figure 8).

Colistin played a critical role in managing MDR infections during the pandemic, while pathogens such as *A. baumannii* and *K. pneumoniae* continue to pose significant challenges. However, the weak link between colistin dose and survival emphasizes the importance of developing new antibiotics and optimizing treatment protocols.

**Figure 9:**
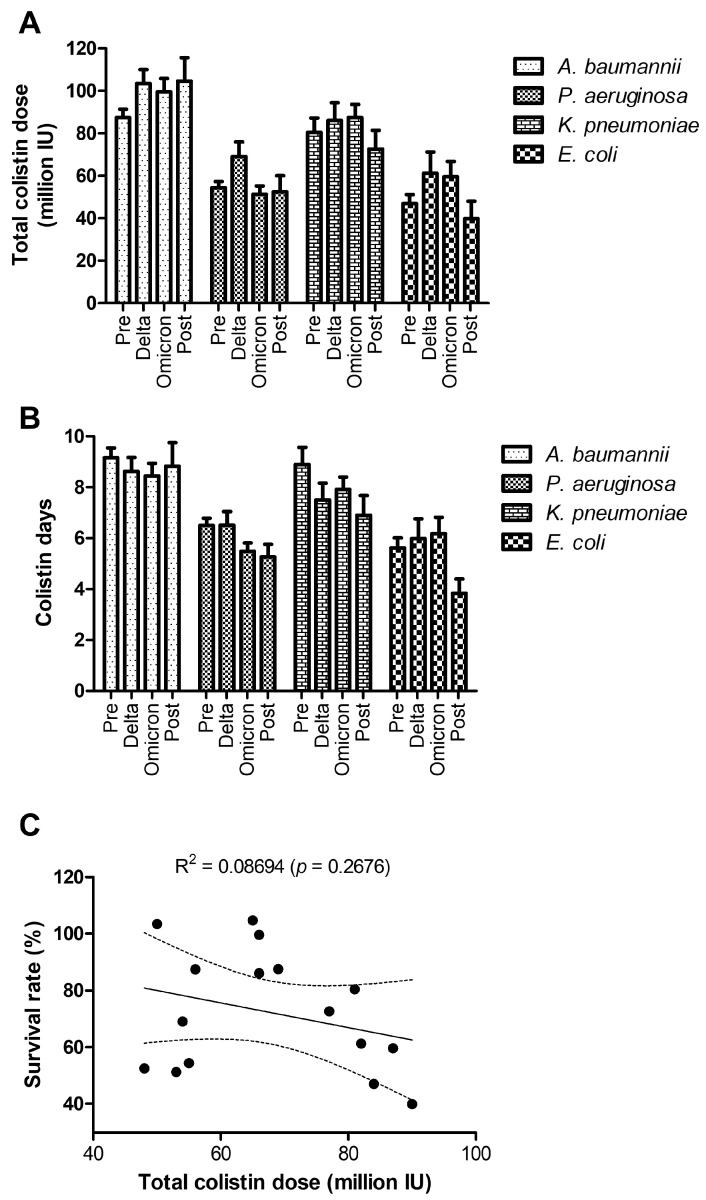
Colistin treatment between 2017 and 2023 during the pre-pandemic, COVID-19 pandemic (Delta, Omicron), and post-pandemic periods: (**A**) Colistin number of vials administered during hospitalization expressed in million IU and (**B**) time of colistin treatments (days)—bars represent the mean ± s.e.m. (**C**) Correlation between colistin consumption and survival rate (Pearson correlation). The linear regression graph shows the values (dots) and the best-fit line with the 95% confidence band.

The Delta pandemic phase was marked by higher colistin doses (Figure 9A) and extended treatment durations (Figure 9B), reflecting the increased burden of severe MDR infections in ICU settings. *A. baumannii* and *K. pneumoniae* stand out as pathogens requiring the most aggressive colistin use, underscoring their resistance profiles and association with poor outcomes. Reductions in treatment durations and doses post-pandemic suggest healthcare systems are regaining stability and managing infections more effectively. The weak correlation between colistin dose and survival rate underscores the complexity of treating MDR infections and highlights the need for additional therapeutic strategies to enhance patient outcomes (Figure 9C).

**Figure 10:**
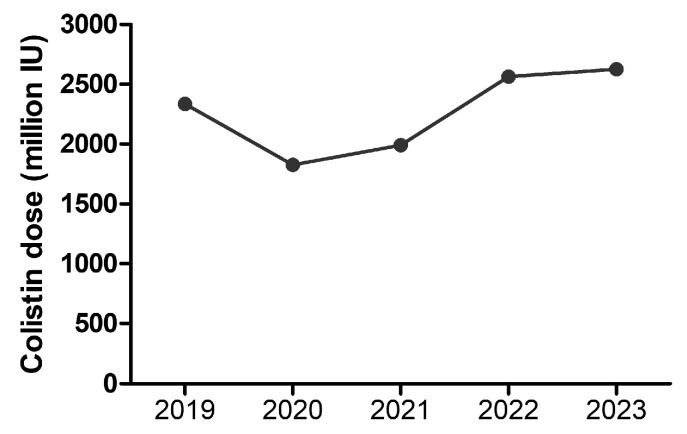
Colistin consumption (number of vials) between 2019 and 2023.

The trend in colistin doses administered every year over the six-year period from 2019 to 2023, providing insights into the fluctuating usage of this critical antibiotic, is illustrated in Figure 10. These variations also reflect changes in clinical practices, healthcare system pressures, and possibly the prevalence of MDR infections during and after the COVID-19 pandemic. By analyzing this trend, we can better understand how external factors, such as pandemics and antimicrobial resistance patterns, influence the reliance on colistin as a treatment option (Figure 10).

**Figure 11:**
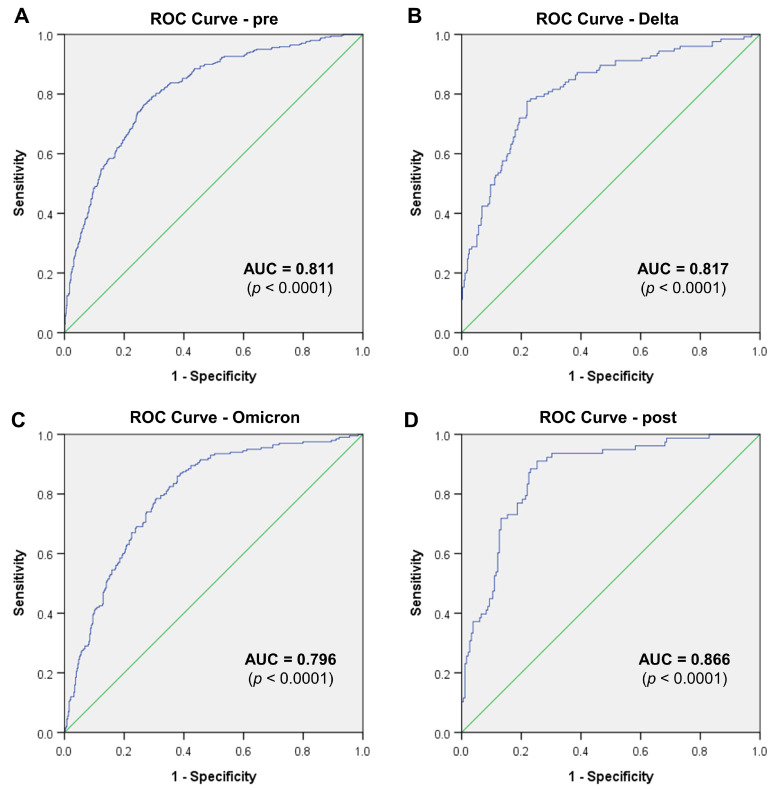
ROC curves predicting mortality generated for the mathematical Model_1 for each period. (**A**) Pre-pandemic; (**B**) Delta; (**C**) Omicron; (**D**) post-pandemic (Model_1 = gender (M), age, hospitalization days, colistin days, *A. baumannii*, *P. aeruginosa*, colistin-resistant GNB, wound culture). The green lines are the diagonal reference lines.

Next, we aimed to identify which clinical or paraclinical factors have the greatest influence on mortality and identify mathematical models that can predict mortality based on our observations. Starting from the data achieved from the AUC analysis of each individual parameter for each pandemic phase, we identified three distinct potential mathematical models (Supplementary Tables S1–S4). Among all described models, Model_1 demonstrated strong predictive accuracy for mortality across different pandemic phases as confirmed by ROC analysis (Figure 11 and Table 2). The consistently high AUC values highlight its clinical utility, with improvements in the post-pandemic period pointing to opportunities for further optimization. The AUC values across all phases (ranging from 0.796 to 0.866) indicate that the model consistently performs well in predicting mortality, regardless of external disruptions. The Delta phase shows slightly better predictive accuracy compared to the pre-pandemic due to heightened mortality risks and reliance on key predictors like hospitalization days and colistin duration. Post-pandemic accuracy improves significantly, reflecting improved healthcare outcomes and management practices. The inclusion of factors like gender, age, hospitalization days, colistin usage, and specific pathogens (*A. baumannii*, *P. aeruginosa*, and resistant GNB) ensures robust model performance (Figure 11).

To further determine the independent predictors for mortality for each pandemic phase, we performed both univariate and multivariate analysis. In the pre-pandemic phase, mortality predictors included (i) age and prolonged colistin treatment (HR > 1, *p* < 0.0001); (ii) pathogen-specific impact, where *A. baumannii* showed the highest hazard ratio (HR 6.786) for mortality, followed by *K. pneumoniae* and *P. aeruginosa*, underscoring the severity of infections caused by these pathogens; and (iii) culture types, where endotracheal aspirates (HR 3.152) were significantly associated with mortality, highlighting the severity of respiratory infections in these patients (Table 3).

In the Delta period, *A. baumannii* remained a major predictor of mortality (HR 5.548); SARS-CoV-2 co-infection emerged as a significant risk factor (HR 1.827, *p* < 0.05), reflecting the compounded risk of COVID-19 in these patients; and colistin dose and treatment duration had less consistent associations with outcomes, likely due to varying treatment protocols during the pandemic (Table 4). 

During the Omicron period, we observed that *K. pneumoniae* and *E. coli* showed increased hazard ratios compared to the Delta period, indicating a shift in the dominant pathogens or resistance patterns. Additionally, the endotracheal aspirate culture type remained a strong predictor of poor outcomes (HR 5.949, *p* < 0.0001) (Table 5).

Post-Pandemic Period: Key mortality predictors: Age (HR 1.069, *p* < 0.0001) and co-infections with colistin-resistant Gram-negative bacteria (HR 10.087, *p* = 0.001) were the strongest predictors of mortality; prolonged hospital stays were inversely associated with mortality (HR 0.943), possibly reflecting the selective admission of patients likely to survive. *A. baumannii* remained the most lethal pathogen (HR 7.998), followed by *K. pneumoniae* (HR 4.644) (Table 6).

## 3. Discussion

The overuse and improper administration of antibiotics in healthcare systems are major contributors to the development and disseminations of antibiotic-resistant bacteria [32,33,34].

The SARS-CoV-2 pandemic significantly influenced AMR trends, as previously described [35]. Our study confirms that pandemic-related factors, such prolonged hospital stays, higher ventilator use, and excessive empirical antibiotic prescribing, likely contributed to the observed rise in colistin-resistant isolates.

During the first two years of the COVID-19 pandemic (2020–2021), there was a significant increase in the antimicrobial prescriptions and total days of therapy, largely due to increased concerns over secondary bacterial infections, as well as the use of empirical antibiotics, including broad-spectrum agents [36,37]. It is estimated that 80% of hospitalized COVID-19 patients received antibiotics between March and October 2020, according to U.S. reports [2,38].

In our study, each pathogen demonstrates a unique distribution pattern across sample types, reflecting its biological niches and clinical manifestations. Wound cultures and endotracheal aspirates dominate nosocomial pathogens like *A. baumannii* and *P. aeruginosa*, while urine cultures are the main source for *E. coli*. The 2019–2021 period in our study shows notable shifts in the distribution for *A. baumannii* and *K. pneumoniae*, particularly in respiratory and blood cultures. This aligns with increased ICU admissions, invasive procedures, and ventilator use during the pandemic.

The present study highlights critical trends in the management of GNB infections over a decade, focusing on the impact of colistin therapy during and after the COVID-19 pandemic. Significant findings include the increased frequency of colistin use during the pandemic, with notable spikes in the Delta and Omicron phases, reflecting the growing prevalence of multidrug-resistant (MDR) infections; persistent challenges with *A. baumannii* and *K. pneumoniae*, which were the most frequent pathogens associated with colistin resistance and mortality, particularly during the pandemic. The proposed mathematical Model_1 demonstrated strong predictive capabilities for mortality across pandemic phases, with the highest accuracy during the post-pandemic period (AUC = 0.866).

The findings underscore the critical role of colistin in managing MDR GNB infections when therapeutic options are limited. The significant increase in colistin use during the pandemic highlights its essential role in treating severe infections caused by pathogens such as *A. baumannii* and *K. pneumoniae*. However, the rise in colistin-resistant infections during this period presents an alarming challenge for clinicians; during the Delta phase, the prevalence of *A. baumannii* and associated mortality (HR = 5.548, *p* < 0.0001) was particularly concerning, reflecting its role in severe cases. The Omicron phase saw an increase in *K. pneumoniae*-related infections, with colistin resistance further complicating management.

The study of Medrzycka-Dabrowska et al. on global antimicrobial resistance from 2021 has identified *A. baumannii* and *K. pneumoniae* as major contributors to MDR infections worldwide [39]. Consistent with these findings, our study also observed significant mortality associated with these pathogens, particularly among critically ill patients.

*A. baumannii* exhibited the highest mortality rates at various time points, highlighting the need for greater attention to bloodstream infections caused by this antibiotic-resistant bacterium. Additionally, a study reported 30-day all-cause mortality rates of 54.7%, 80.5%, and 63.8% for bacteremia caused by carbapenem-resistant *A. baumannii*, *P. aeruginosa*, and *K. pneumoniae*, respectively [40].

We observed a higher incidence of *A. baumannii* infections during the pandemic, similar to the findings reported by Cogliati Dezza et al., thus aligning with other reports that found carbapenem-resistant *A. baumannii* as the dominant pathogen. Its ability to persist in hospital environments and thrive in immunocompromised patients contributed to its increased prevalence. Their study further highlighted a notable shift in the epidemiology of MDR GNB, with a rise in CR *A. baumannii* infections in hospital admissions, while bloodstream infections caused by carbapenem-resistant *K. pneumoniae* (CR-KP) declined [35].

Our results similarly suggest that colistin-resistant isolates were predominantly *A. baumannii*, reinforcing concerns about its persistence as a difficult-to-treat pathogen.

Russo et al. further confirmed that COVID-19 patients had a higher incidence of MDR-AB infections, attributing this increase to prolonged mechanical ventilation and excessive antibiotic use [41].

Alenazi et al. demonstrated that COVID-19 patients co-infected with MDR-AB had significantly higher mortality rates than non-COVID-19 patients. They concluded that MDR-AB infections worsen prognosis in critically ill patients, which is consistent with our findings that colistin-resistant GNB infections were associated with prolonged hospitalization and poor clinical outcomes [42].

Patients with CR *A. baumannii* infections demonstrated significantly worse survival rates, as reported in our study as well as in the above-mentioned studies by Cogliati Dezza et al. and Alenazi et al. These findings highlight the urgent need for alternative therapeutic options beyond colistin. In a meta-analysis of 24 studies examining bacterial co-infections in hospitalized COVID-19 patients, co-infection rates were reported at 3.5% (95% CI: 0.4–6.7%) and secondary infections at 14.3% (95% CI: 9.6–18.9%) [43]. Bacterial co-infections were observed in 6.9% of cases, ranging from 5.9% in general hospitalized patients to 8.1% in critically ill individuals [43]. Common co-pathogens included *A. baumannii*, *K. pneumoniae*, *E. coli*, *P. aeruginosa*, *Streptococcus pneumoniae*, *S. aureus*, and others, including fungal species like *Candida* and *Aspergillus*, and viral pathogens such as influenza and rhinovirus [23,44,45,46,47]. These findings highlight the diversity of co-pathogens complicating COVID-19 cases, particularly in critically ill patients.

The findings of our research indicate that Gram-negative isolates were predominant among hospitalized COVID-19 patients, aligning with the study by Bazaid A.S. et al., which identified Gram-negative bacteria as the most common pathogens in COVID-19 cases [48].

Furthermore, the analysis reveals a rising trend in *A. baumannii* co-infections over the years, with the highest frequencies recorded between 2017 and 2021. The differences were statistically significant (*p* < 0.0001). These results highlight the prominent role of *A. baumannii*, a highly resistant pathogen, in severe polymicrobial infections, particularly during the pandemic years when antibiotic usage and hospitalization rates surged.

The results of our study demonstrate that *A. baumannii* and *K. pneumoniae* were the most frequently isolated bacterial species, particularly from sputum and blood samples. Supporting these results, *A. baumannii* has been identified as a primary pathogen in the respiratory tracts of COVID-19 patients, accounting for approximately 10% of all cultured samples [23]. Furthermore, another study reported its presence in 90% of COVID-19 patients [41]. Coinfection with *A. baumannii* in COVID-19 patients has been significantly associated with the development of systemic infections and an increased risk of mortality [22,49]. These findings are also consistent with the results of our study, highlighting the critical impact of *A. baumannii* on patient outcomes.

Similar trends are seen with other co-infections, especially during the years surrounding 2020. *P. aeruginosa* and *K. pneumoniae* combinations are particularly frequent, with statistical significance (*p* < 0.0001). This highlights the burden of multidrug-resistant GNB as co-pathogens during periods of increased healthcare strain, such as the pandemic.

Medrzycka-Dabrowska et al. reported in a recent review that the prevalence of carbapenem-resistant *K. pneumoaniae* isolated among COVID-19 patients ranged from 0.35% to 53% across six countries—Italy, China, Egypt, the United States, Spain, and Peru [39]. Additionally, *P. aeruginosa* has been frequently identified as a common co-infecting pathogen in COVID-19 patients, contributing to the exacerbation of illness [50,51,52,53,54].

In a study documenting antimicrobial use (AU), the consumption of colistin, imipenem, and meropenem significantly increased in the ICU and general wards after the onset of COVID-19 while remaining stable in the emergency department [55]. Our study similarly observed an increase in colistin use during the pandemic, particularly in critically ill patients. However, unlike the referenced study, our data further highlight a distinct spike in colistin use during the Omicron phase, reflecting the ongoing reliance on this antibiotic in managing multidrug-resistant infections during the pandemic’s later stages. This comparison underscores the consistent pattern of heightened colistin use in response to the pandemic’s pressures on healthcare systems, especially in settings managing severe infections.

Our findings expand on prior research by providing a decade-long perspective that incorporates the unique pressures of the COVID-19 pandemic. Notably, the significant increase in colistin use during the pandemic is consistent with studies that have reported heightened antimicrobial consumption due to prolonged hospital stays, increased ventilator usage, and co-infections. Our study also provides novel insights into the differential impacts of pandemic phases on pathogen prevalence in each sample type.

The longitudinal design of our study is a significant strength, providing a comprehensive analysis of trends over a decade and capturing both pre-pandemic and pandemic-specific dynamics. Additionally, the use of predictive modeling (e.g., Model_1) offers valuable insights into mortality risks and clinical outcomes, with important implications for patient management.

The limitations of our study include its retrospective design, which may introduce selection bias due to the focus on colistin-treated patients in specific settings, and its restricted temporal and geographic scope, which may not fully capture global trends. Additionally, the analysis is limited to a single center in Romania, which while representative of the northeastern region of the country, may not fully capture variations in clinical practices, healthcare infrastructure, and patient demographics across different regions. Expanding the study to include multiple centers would enhance the robustness of our findings, allowing for a more comprehensive understanding of GNB infections, treatment responses, and potential regional differences in healthcare delivery. This broader approach would ultimately strengthen the validity and generalizability of our conclusions.

## 4. Materials and Methods

We conducted a retrospective observational study to evaluate trends in colistin resistance and clinical outcomes among colistin-treated patients over a 13-year period (2011–2023).

The study was conducted at “Saint Spiridon” County Clinical Emergency Hospital, a tertiary care facility in Iasi, Romania, and focused on Gram-negative bacterial (GNB) infections, particularly those caused by *Klebsiella pneumoniae*, *Acinetobacter baumannii*, *Pseudomonas aeruginosa*, and *Escherichia coli*. Notably, “Saint Spiridon” Emergency Clinical County Hospital serves as a university hospital that, according to internal statistical data, provides medical care to 67% of patients from Iasi County and 33% of patients from the northeastern region of Romania. This region has a population of over 3.7 million residents, accounting for more than 17% of the country’s total population [24]. The study period was further divided into three phases, namely the pre-pandemic (2011–2019), pandemic (Delta and Omicron waves, 2020–2022), and post-pandemic (2023).

In Romania, the use of colistin is guided by national and international antimicrobial stewardship recommendations, particularly in the treatment of multidrug-resistant (MDR) Gram-negative infections. The national guidelines, issued by the Romanian Society of Infectious Diseases, align with the European Committee on Antimicrobial Susceptibility Testing (EUCAST) and European Society of Clinical Microbiology and Infectious Diseases (ESCMID) guidelines, which emphasize colistin as a last-resort antibiotic for carbapenem-resistant *A. baumannii*, *P. aeruginosa*, and *K. pneumoniae* infections [56,57]. The national guidelines recommend intravenous colistin use only in cases where other therapeutic options are ineffective, with dosing adjusted based on renal function to minimize nephrotoxicity. The final therapeutic decision was left at the discretion of the attending physicians.

### 4.1. Isolate Characterization

Bacterial isolates were identified using standard microbiological techniques and using matrix-assisted laser desorption ionization–time-of-flight mass spectrometry (MALDI-TOF MS) (Bruker Daltonik GmbH, Bremen, Germany).

Colistin susceptibility testing was performed by the broth microdilution method in an automated system on the MICRONAUT-S (Merlin, Dortmund, Germany) and by broth microdilution strips. For the other antibiotics, testing was conducted using both the MICRONAUT-S system and the disk diffusion method. The interpretation of the results was performed according to the European Committee on Antimicrobial Susceptibility Testing (EUCAST) standard, applicable at the time [57]. For colistin, The Clinical and Laboratory Standards Institute (CLSI) and EUCAST have established colistin susceptibility testing through the determination of the minimal inhibitory concentration (MIC) using broth microdilution, a standardized method worldwide accepted [57,58].

### 4.2. Statistical Analysis

Data analysis was conducted using Graph Pad Prism, v5 (Graph Pad Software, San Diego, CA, USA) and SPSS, v25 (IBM SPSS Software, Chicago, IL, USA). The significant associations between distinct categorical variables were determined using the chi-squared test. Univariate and multivariate analyses were performed to identify factors associated with patient mortality. Hazard ratios (HRs) and 95% confidence intervals (CIs) were calculated for key clinical and paraclinical variables. Receiver operating characteristic (ROC) curves were generated to assess the predictive accuracy of the models for mortality across different phases of the pandemic. The pandemic period was temporally split in four phases, namely the pre-pandemic (from 1 January 2017 until the 25 February 2020), the Delta pandemic time (from the start of SARS-CoV-2 pandemic on the 26 February 2020 until the 3 December 2021), the Omicron period (from the 4 December 2021, the date when the first two cases of Omicron infection were officially reported in Romania, until the 5 May 2023), and post-pandemic (starting with 5 May 2023 until the end of 2024). Statistical significance was defined as *p* < 0.05.

## 5. Conclusions

This study highlights the evolving landscape of GNB infections and colistin resistance over the past decade, with the COVID-19 pandemic serving as a pivotal factor in shaping treatment and resistance patterns. The findings point to the importance of continued antibiotic resistance monitoring, along with trend analysis, to better understand the evolution of MDR infections.

## Figures and Tables

**Table 1:** General characteristics of patients.

	Total, N %	F, n % M, n %	Age Median [CI]
**2011**	393.00 (7.73)	177 (45.04) 216 (54.96)	65 [55–74]
**2012**	375.00 (7.38)	148 (39.47) 227 (60.53)	63 [54–74]
**2013**	437.00 (8.60)	151 (34.55) 286 (65.45)	63 [52.5–75]
**2014**	502.00 (9.88)	185 (36.85) 317 (63.15)	65 [54–75]
**2015**	423.00 (8.33)	158 (37.35) 265 (62.65)	65 [57–74]
**2016**	420.00 (8.27)	144 (34.29) 276 (65.71)	65 [55–74]
**2017**	370.00 (7.28)	140 (37.84) 230 (62.16)	64 [53–75]
**2018**	417.00 (8.21)	149 (35.73) 268 (64.27)	64 [53–73]
**2019**	358.00 (7.05)	144 (40.22) 214 (59.78)	64 [52–72]
**2020**	247.00 (4.86)	98 (39.68) 149 (60.32)	63 [52–73]
**2021**	304.00 (5.98)	110 (36.18) 194 (63.82)	63 [53–74]
**2022**	431.00 (8.48)	148 (34.34) 283 (65.66)	65 [54–73]
**2023**	404.00 (7.95)	157 (38.86) 247 (61.14)	66 [54–73]

Abbreviations: N = total number; F = female; M = male; n = number; CI = confidence interval. The smallest N values are highlighted in red.

**Table 2:** ROC evaluation of distinct mathematical models based on paraclinical and clinical parameters in association with mortality for each pandemic phase.

Model	AUC	S.E.	*p*-Value	95% CI
*Pre-pandemic*				
**Model_1**	0.811	0.014	<0.0001	0.785–0.838
**Model_2**	0.756	0.015	<0.0001	0.726–0.786
**Model_3**	0.793	0.014	<0.0001	0.765–0.821
*Delta*				
**Model_1**	0.817	0.023	<0.0001	0.773–0.862
**Model_2**	0.785	0.024	<0.0001	0.738–0.832
**Model_3**	0.800	0.025	<0.0001	0.751–0.849
*Omicron*				
**Model_1**	0.796	0.019	<0.001	0.758–0.833
**Model_2**	0.772	0.020	<0.001	0.732–0.811
**Model_3**	0.772	0.020	<0.001	0.733–0.811
*Post-pandemic*				
**Model_1**	0.866	0.024	<0.0001	0.819–0.912
**Model_2**	0.836	0.026	<0.0001	0.785–0.887
**Model_3**	0.847	0.024	<0.0001	0.800–0.895

Abbreviations: AUC = area under curve; *p* = statistical significance coefficient; M = male; CI = confidence interval; Model_1 = gender (M), age, hospitalization days, colistin days, *A. baumannii*, *P. aeruginosa*, colistin-resistant GNB, wound culture; Model_2 = gender (M), age, hospitalization days, colistin days, colistin-resistant GNB, wound culture, abscess culture, SARS-CoV-2 infection; Model_3 = gender (M), hospitalization days, colistin dose, colistin days, *A. baumannii*, *P. aeruginosa*, colistin-resistant GNB, wound culture, SARS-CoV-2 infection.

**Table 3:** Univariate and multivariate mortality regression analysis of paraclinical and clinical variables in colistin-treated patients during the pre-pandemic period.

Variable (Pre)	Univariate Analysis			Multivariate Analysis		
	HR	95% CI	*p*-Value	HR	95% CI	*p*-Value
Gender (M)	0.899	0.655–1.232	0.509			
Age	1.036	1.024–1.047	**<0.0001**	1.037	1.025–1.048	**<0.0001**
Hospitalization days	0.960	0.948–0.972	**<0.0001**	0.960	0.947–0.972	**<0.0001**
Colistin dose	0.996	0.989–1.002	0.185			
Colistin days	1.117	1.045–1.193	**0.001**	1.077	1.044–1.110	**<0.0001**
*A. baumannii*	7.083	4.637–10.81	**<0.0001**	6.786	4.495–10.245	**<0.0001**
*P. aeruginosa*	2.367	1.585–3.534	**<0.0001**	2.240	1.516–3.310	**<0.0001**
*K. pneumoniae*	3.045	2.046–4.530	**<0.0001**	3.023	2.037–4.483	**<0.0001**
*E. coli*	1.890	1.166–3.060	**0.010**	1.889	1.171–3.045	**0.009**
Co-infection with colistin-resistant GNB	1.690	1.050–2.719	**0.031**	1.626	1.016–2.600	**0.043**
Wound culture	0.439	0.228–0.842	**0.013**	0.315	0.214–0.463	**<0.0001**
Endotracheal aspirate	4.552	2.290–9.047	**<0.0001**	3.152	2.077–4.782	**<0.0001**
Urine culture	0.861	0.352–2.105	0.743			
Blood culture	2.418	0.957–6.108	0.062			
Fluid culture	2.300	0.983–5.382	0.055			
Abscess culture	1.195	0.430–3.312	0.732			

Abbreviations: M = male; GNB = Gram-negative bacteria; HR = hazard ratio; CI = confidence interval; *p* = statistical significance coefficient; significant *p*-values are highlighted in bold.

**Table 4:** Univariate and multivariate mortality regression analysis of paraclinical and clinical variables in colistin-treated patients during the Delta pandemic period.

Variable (Delta)	Univariate Analysis			Multivariate Analysis		
	HR	95% CI	*p*-Value	HR	95% CI	*p*-Value
Gender (M)	0.938	0.555–1.584	0.811			
Age	1.037	1.018–1.056	**<0.0001**	1.027	1.010–1.043	**0.001**
Hospitalization days	0.953	0.933–0.971	**<0.0001**	0.975	0.961–0.988	**<0.0001**
Colistin dose	1.001	0.992–1.009	0.811			
Colistin days	1.091	0.989–1.203	0.081			
*A. baumannii*	6.620	3.344–13.102	**<0.0001**	5.548	3.070–10.024	**<0.0001**
*P. aeruginosa*	2.046	1.086–3.850	**0.027**	1.709	0.997–2.926	0.051
*K. pneumoniae*	1.444	0.807–2.579	0.215			
*E. coli*	1.357	0.610–3.017	0.454			
Co-infection with colistin-resistant GNB	1.477	0.656–3.324	0.346			
Wound culture	0.534	0.129–2.206	0.386			
Endotracheal aspirate	4.369	1.029–18.533	**0.046**	6.159	3.499–10.838	**<0.0001**
Urine culture	0.677	0.112–4.087	0.671			
Blood culture	3.683	0.689–19.662	0.127			
Fluid culture	3.823	0.692–21.109	0.124			
Abscess culture	0.198	0.016–2.378	0.202			
SARS-CoV-2 infection confirmed	2.011	1.046–3.864	**0.036**	1.827	1.002–3.346	0.049

Abbreviations: M = male; GNB = Gram-negative bacteria; HR = hazard ratio; CI = confidence interval; *p* = statistical significance coefficient; significant *p*-values are highlighted in bold.

**Table 5:** Univariate and multivariate mortality regression analysis of paraclinical and clinical variables in colistin-treated patients during the Omicron pandemic period.

Variable (Omicron)	Univariate Analysis			Multivariate Analysis		
	HR	95% CI	*p*-Value	HR	95% CI	*p*-Value
Gender (M)	0.854	0.537–1.356	0.504			
Age	1.044	1.027–1.060	**<0.0001**	1.031	1.014–1.048	**<0.0001**
Hospitalization days	0.952	0.935–0.968	**<0.0001**	0.954	0.935–0.972	**<0.0001**
Colistin dose	1.011	1.004–1.018	**0.002**	1.008	1.004–1.012	**<0.0001**
Colistin days	1.032	0.955–1.113	0.424			
*A. baumannii*	3.365	1.812–6.250	**<0.0001**	3.809	2.214–6.551	**<0.0001**
*P. aeruginosa*	1.242	0.692–2.227	0.468			
*K. pneumoniae*	4.731	2.672–8.374	**<0.0001**	1.091	0.649–1.833	0.742
*E. coli*	4.091	2.029–8.245	**<0.0001**	1.000	0.496–2.010	0.999
Co-infection with colistin-resistant GNB	1.867	0.814–4.282	0.140			
Wound culture	0.602	0.208–1.738	0.348			
Endotracheal aspirate	12.399	3.858–39.842	**<0.0001**	5.949	3.358–10.535	**<0.0001**
Urine culture	0.776	0.218–2.752	0.695			
Blood culture	2.043	0.613–6.805	0.245			
Fluid culture	1.232	0.327–4.628	0.758			
Abscess culture	2.066	0.422–10.093	0.370			
SARS-CoV-2 infection confirmed	1.125	0.471–2.683	0.790			

Abbreviations: M = male; GNB = Gram-negative bacteria; HR = hazard ratio; CI = confidence interval; *p* = statistical significance coefficient; significant *p*-values are highlighted in bold.

**Table 6:** Univariate and multivariate mortality regression analysis of paraclinical and clinical variables in colistin-treated patients during the post-pandemic period.

Variable (Post).	Univariate Analysis			Multivariate Analysis		
	HR	95% CI	*p*-Value	HR	95% CI	*p*-Value
Gender (M)	1.064	0.469–2.410	0.882			
Age	1.072	1.038–1.107	**<0.0001**	1.069	1.037–1.101	**<0.0001**
Hospitalization days	0.943	0.910–0.975	**0.001**	0.943	0.913–0.973	**<0.0001**
Colistin dose	1.016	1.003–1.027	**0.012**	1.008	1.002–1.014	**0.007**
Colistin days	0.911	0.795–1.043	0.177			
*A. baumannii*	11.610	3.255-	**<0.0001**	7.998	3.368–18.987	**<0.0001**
*P. aeruginosa*	1.479	0.452–4.835	0.517			
*K. pneumoniae*	6.368	2.104–19.263	**0.001**	4.644	1.967–10.959	**<0.0001**
*E. coli*	1.800	0.439–7.372	0.414			
Co-infection with colistin-resistant GNB	7.860	1.774–34.816	**0.007**	10.087	2.654–38.329	**0.001**
Wound culture	0.099	0.024–0.394	**0.001**	0.105	0.046–0.233	**<0.0001**
Endotracheal aspirate	0.887	0.229–3.427	0.862			
Urine culture	0.505	0.081–3.127	0.463			
Blood culture	2.361	0.432–12.890	0.321			
Fluid culture	1.437	0.189–10.880	0.726			
Abscess culture	2.392	0.135–42.221	0.552			
SARS-CoV-2 infection confirmed	6.623	0.834–52.545	0.074			

Abbreviations: M = male; GNB = Gram-negative bacteria; HR = hazard ratio; CI = confidence interval; *p* = statistical significance coefficient; significant *p*-values are highlighted in bold.

## Data Availability

The data are contained within the article. Further inquiries can be directed to the corresponding author.

## Supplementary Materials

The following supporting information can be downloaded at: https://www.mdpi.com/article/10.3390/antibiotics14030275/s1, Table S1: ROC evaluation of paraclinical and clinical parameters in association with mortality in the pre-pandemic period; Table S2: ROC evaluation of paraclinical and clinical parameters in association with mortality in the Delta pandemic period; Table S3: ROC evaluation of paraclinical and clinical parameters in association with mortality in the Omicron pandemic period; Table S4: ROC evaluation of paraclinical and clinical parameters in association with mortality in the post-pandemic period.
