# Supplementary material for: Colistin Treatment Outcomes in Gram-Negative Bacterial Infections in the Northeast of Romania: A Decade of Change Through Pandemic Challenges
Source: Antibiotics (Basel). 2025 Mar 7;14(3):275. doi: 10.3390/antibiotics14030275 (PMC11939382; doi:10.3390/antibiotics14030275)
Supplement: Supplementary file 1 [file antibiotics-14-00275-s001.zip › antibiotics-3480941-supplementary.pdf]

**Supplementary Table S1.** **ROC evaluation of paraclinical and clinical parameters in association with mortality in the pre-pandemic period.**

| Analyte | AUC | S.E. | *p*-value | 95% Confidence Interval | Cut-off value | Sensitivity | Specificity |
|---|---|---|---|---|---|---|---|
| Gender (M) | 0.589 | 0.018 | **< 0.0001** | 0.553-0.623 | | | |
| Age | 0.456 | 0.017 | **0.017** | 0.421-0.490 | 67.50 | 0.475 | 0.642 |
| Hospitalization days | 0.577 | 0.017 | **< 0.0001** | 0.543-0.610 | 15.50 | 0.637 | 0.385 |
| Colistin dose | 0.530 | 0.019 | 0.109 | 0.492-0.566 | | | |
| Colistin days | **0.643** | 0.018 | **< 0.0001** | 0.608-0.677 | 6.50 | 0.407 | 0.672 |
| *A. baumannii* | 0.459 | 0.018 | **0.027** | 0.423-0.495 | | | |
| *P. aeruginosa* | 0.543 | 0.019 | **0.020** | 0.505-0.579 | | | |
| *K. pneumoniae* | 0.473 | 0.018 | 0.145 | 0.437-0.508 | | | |
| *E. coli* | 0.497 | 0.018 | 0.876 | 0.460-0.533 | | | |
| Co-infection with colistin-resistant GNB | **0.331** | 0.017 | **< 0.0001** | 0.296-0.364 | | | |
| Wound culture | **0.674** | 0.019 | **< 0.0001** | 0.637-0.710 | | | |
| Endotracheal aspirate | 0.481 | 0.018 | 0.304 | 0.445-0.516 | | | |
| Urine culture | 0.505 | 0.019 | 0.795 | 0.468-0.541 | | | |
| Blood culture | 0.514 | 0.019 | 0.456 | 0.477-0.550 | | | |
| Fluid culture | 0.500 | 0.018 | 0.983 | 0.463-0.535 | | | |
| Abscess culture | 0.500 | 0.018 | 1.000 | 0.463-0.536 | | | |

Abbreviations: AUC = area under curve; *p* = statistical significance coefficient; M = male; Model_1 = gender(M), age, hospitalization days, colistin days, *A. baumannii*, *P. aeruginosa*, Colistin-resistant GNB, wound culture; Model_2 = gender(M), age, hospitalization days, colistin days, Colistin-resistant GNB, wound culture, abscess culture, SARS-CoV-2 infection; Model_3 = gender(M), hospitalization days, colistin dose, colistin days, *A. baumannii*, *P. aeruginosa*, Colistin-resistant GNB, wound culture, SARS-CoV-2 infection.

**Supplementary Table S2.** **ROC evaluation of paraclinical and clinical parameters in association with mortality in the Delta pandemic period.**

| **Analyte** | **AUC** | **S.E.** | ***p*-value** | **95% Confidence Interval** | **Cut-off value** | **Sensitivity** | **Specificity** |
|---|---|---|---|---|---|---|---|
| Gender (M) | 0.614 | 0.030 | **< 0.0001** | 0.555-0.671 | | | |
| Age | 0.422 | 0.029 | **0.009** | 0.365-0.477 | 64.5 | 0.616 | 0.587 |
| Hospitalization days | 0.594 | 0.027 | **0.002** | 0.540-0.647 | 16.50 | 0.592 | 0.336 |
| Colistin dose | 0.544 | 0.031 | 0.141 | 0.483-0.604 | | | |
| Colistin days | 0.623 | 0.029 | **< 0.0001** | 0.567-0.679 | 5.50 | 0.472 | 0.601 |
| *A. baumannii* | 0.476 | 0.030 | 0.416 | 0.416-0.534 | | | |
| *P. aeruginosa* | 0.492 | 0.030 | 0.781 | 0.432-0.550 | | | |
| *K. pneumoniae* | 0.467 | 0.029 | 0.275 | 0.409-0.524 | | | |
| *E. coli* | 0.492 | 0.030 | 0.795 | 0.433-0.550 | | | |
| Co-infection with colistin-resistant GNB | 0.325 | 0.029 | **< 0.0001** | 0.269-0.381 | | | |
| Wound culture | 0.671 | 0.031 | **< 0.0001** | 0.610-0.730 | | | |
| Endotracheal aspirate | 0.483 | 0.030 | 0.578 | 0.425-0.541 | | | |
| Urine culture | 0.526 | 0.031 | 0.392 | 0.465-0.585 | | | |
| Blood culture | 0.522 | 0.031 | 0.470 | 0.461-0.581 | | | |
| Fluid culture | 0.484 | 0.030 | 0.596 | 0.425-0.542 | | | |
| Abscess culture | 0.564 | 0.031 | **0.035** | 0.502-0.624 | | | |
| SARS-CoV-2 infection confirmed | 0.614 | 0.030 | **< 0.0001** | 0.555-0.671 | | | |

Abbreviations: AUC = area under curve; *p* = statistical significance coefficient; M = male; Model_1 = gender(M), age, hospitalization days, colistin days, *A. baumannii*, *P. aeruginosa*, Colistin-resistant GNB, wound culture; Model_2 = gender(M), age, hospitalization days, colistin days, Colistin-resistant GNB, wound culture, abscess culture, SARS-CoV-2 infection; Model_3 = gender(M), hospitalization days, colistin dose, colistin days, *A. baumannii*, *P. aeruginosa*, Colistin-resistant GNB, wound culture, SARS-CoV-2 infection.

**Supplementary Table S3.** **ROC evaluation of paraclinical and clinical parameters in association with mortality in the Omicron pandemic period.**

| **Analyte** | **AUC** | **S.E.** | ***p*-value** | **95% Confidence Interval** | **Cut-off value** | **Sensitivity** | **Specificity** |
|---|---|---|---|---|---|---|---|
| Gender (M) | 0.567 | 0.025 | **0.008** | 0.517-0.615 | | | |
| Age | 0.479 | 0.024 | 0.399 | 0.431-0.526 | | | |
| Hospitalization days | 0.678 | 0.022 | **< 0.0001** | 0.635-0.720 | 16.50 | 0.610 | 0.405 |
| Colistin dose | 0.614 | 0.024 | **< 0.0001** | 0.565-0.661 | 33.50 | 0.735 | 0.556 |
| Colistin days | 0.580 | 0.025 | **0.002** | 0.530-0.629 | 6.50 | 0.450 | 0.741 |
| *A. baumannii* | 0.405 | 0.025 | **< 0.0001** | 0.356-0.453 | | | |
| *P. aeruginosa* | 0.583 | 0.025 | **0.001** | 0.533-0.632 | | | |
| *K. pneumoniae* | 0.523 | 0.025 | 0.363 | 0.473-0.572 | | | |
| *E. coli* | 0.489 | 0.025 | 0.669 | 0.439-0.538 | | | |
| Co-infection with colistin-resistant GNB | 0.340 | 0.024 | **< 0.0001** | 0.292-0.387 | | | |
| Wound culture | 0.644 | 0.026 | **< 0.0001** | 0.593-0.693 | | | |
| Endotracheal aspirate | 0.485 | 0.025 | 0.557 | 0.436-0.534 | | | |
| Urine culture | 0.521 | 0.026 | 0.411 | 0.470-0.570 | | | |
| Blood culture | 0.510 | 0.025 | 0.696 | 0.460-0.559 | | | |
| Fluid culture | 0.508 | 0.025 | 0.744 | 0.458-0.557 | | | |
| Abscess culture | 0.499 | 0.025 | 0.977 | 0.449-0.548 | | | |
| SARS-CoV-2 infection confirmed | 0.567 | 0.025 | **0.008** | 0.517-0.615 | | | |

Abbreviations: AUC = area under curve; *p* = statistical significance coefficient; M = male; Model_1 = gender(M), age, hospitalization days, colistin days, *A. baumannii*, *P. aeruginosa*, Colistin-resistant GNB, wound culture; Model_2 = gender(M), age, hospitalization days, colistin days, Colistin-resistant GNB, wound culture, abscess culture, SARS-CoV-2 infection; Model_3 = gender(M), hospitalization days, colistin dose, colistin days, *A. baumannii*, *P. aeruginosa*, Colistin-resistant GNB, wound culture, SARS-CoV-2 infection.

**Supplementary Table S4. ROC evaluation of paraclinical and clinical parameters in association with mortality in the post-pandemic period.**

| **Analyte** | **AUC** | **S.E.** | ***p*-value** | **95% Confidence Interval** | **Cut-off value** | **Sensitivity** | **Specificity** |
|---|---|---|---|---|---|---|---|
| Gender (M) | 0.598 | 0.038 | **0.012** | 0.524-0.672 | | | |
| Age | 0.513 | 0.037 | 0.749 | 0.440-0.584 | | | |
| Hospitalization days | 0.685 | 0.033 | **< 0.0001** | 0.620-0.749 | 12.50 | 0.769 | 0.429 |
| Colistin dose | 0.596 | 0.038 | **0.015** | 0.521-0.670 | 32.00 | 0.769 | 0.604 |
| Colistin days | 0.637 | 0.039 | **< 0.0001** | 0.561-0.712 | 3.50 | 0.667 | 0.516 |
| *A. baumannii* | 0.361 | 0.037 | **< 0.0001** | 0.287-0.433 | | | |
| *P. aeruginosa* | 0.594 | 0.040 | **0.016** | 0.516-0.672 | | | |
| *K. pneumoniae* | 0.505 | 0.039 | 0.907 | 0.427-0.581 | | | |
| *E. coli* | 0.524 | 0.040 | 0.543 | 0.445-0.601 | | | |
| Co-infection with colistin-resistant GNB | 0.260 | 0.034 | **< 0.0001** | 0.192-0.327 | | | |
| Wound culture | 0.605 | 0.040 | **0.007** | 0.526-0.683 | | | |
| Endotracheal aspirate | 0.513 | 0.039 | 0.743 | 0.435-0.590 | | | |
| Urine culture | 0.548 | 0.040 | 0.224 | 0.468-0.626 | | | |
| Blood culture | 0.527 | 0.040 | 0.483 | 0.449-0.605 | | | |
| Fluid culture | 0.510 | 0.039 | 0.797 | 0.432-0.587 | | | |
| Abscess culture | 0.521 | 0.040 | 0.591 | 0.443-0.598 | | | |
| SARS-CoV-2 infection confirmed | 0.598 | 0.038 | **0.012** | 0.524-0.672 | | | |

Abbreviations: AUC = area under curve; *p* = statistical significance coefficient; M = male; Model_1 = gender(M), age, hospitalization days, colistin days, *A. baumannii*, *P. aeruginosa*, Colistin-resistant GNB, wound culture; Model_2 = gender(M), age, hospitalization days, colistin days, Colistin-resistant GNB, wound culture, abscess culture, SARS-CoV-2 infection; Model_3 = gender(M), hospitalization days, colistin dose, colistin days, *A. baumannii*, *P. aeruginosa*, Colistin-resistant GNB, wound culture, SARS-CoV-2 infection.
